# Prokaryotes of renowned Karlovy Vary (Carlsbad) thermal springs: phylogenetic and cultivation analysis

**DOI:** 10.1186/s40793-022-00440-2

**Published:** 2022-09-11

**Authors:** Tereza Smrhova, Kunal Jani, Petr Pajer, Gabriela Kapinusova, Tomas Vylita, Jachym Suman, Michal Strejcek, Ondrej Uhlik

**Affiliations:** 1 Department of Biochemistry and Microbiology, Faculty of Food and Biochemical Technology, University of Chemistry and Technology, Prague, Technicka 3, 166 28 Prague 6, Czech Republic; 2 Military Health Institute, Ministry of Defence of the Czech Republic, Prague, Czech Republic; 3 Institute of Balneology and Spa Sciences, Karlovy Vary, Czech Republic

**Keywords:** Amplicon sequencing analysis, Cultivation analysis, Thermal water springs, Phylogenetic novelty

## Abstract

**Background:**

The extreme conditions of thermal springs constitute a unique aquatic habitat characterized by low nutrient contents and the absence of human impacts on the microbial community composition. Thus, these springs may host phylogenetically novel microorganisms with potential use in biotechnology. With this hypothesis in mind, we examined the microbial composition of four thermal springs of the world-renowned spa town of Karlovy Vary (Carlsbad), Czechia, which differ in their temperature and chemical composition.

**Results:**

Microbial profiling using 16S rRNA gene sequencing revealed the presence of phylogenetically novel taxa at various taxonomic levels, spanning from genera to phyla. Many sequences belonged to novel classes within the phyla Hydrothermae, Altiarchaeota, Verrucomicrobia, and TA06. Cultivation-based methods employing oligotrophic media resulted in the isolation of 44 unique bacterial isolates. These include strains that withstand concentrations of up to 12% NaCl_w/v_ in cultivation media or survive a temperature of 100 °C, as well as hitherto uncultured bacterial species belonging to the genera *Thermomonas*, *Paenibacillus*, and *Cellulomonas*. These isolates harbored stress response genes that allow them to thrive in the extreme environment of thermal springs.

**Conclusions:**

Our study is the first to analyze the overall microbial community composition of the renowned Karlovy Vary thermal springs. We provide insight into yet another level of uniqueness of these springs. In addition to their unique health benefits and cultural significance, we demonstrate that these springs harbor phylogenetically distinct microorganisms with unusual life strategies. Our findings open up avenues for future research with the promise of a deeper understanding of the metabolic potential of these microorganisms.

**Supplementary Information:**

The online version contains supplementary material available at 10.1186/s40793-022-00440-2.

## Background

Thermal springs occur all over the world, mostly in places with volcanic activity, and constitute a unique aquatic microbial habitat [1]. This uniqueness is characterized by their low nutrient content, broad geochemical gradients, and the lack of human impact on both their chemical and microbiological composition [2]. Thus, as-yet-unexplored thermal springs represent model ecosystems for investigating subsurface microbial biogeography. Moreover, a certain analogy can be found between the conditions of such ecosystems and those postulated to have been present in early Earth [3]. Deep thermal springs can host hitherto unknown microorganisms that potentially harbor novel biologically active compounds that might be useful for a broad spectrum of biotechnological applications [4, 5]. In particular, they are great candidates for discovering compounds that are biologically active at high temperatures [6, 7]. Furthermore, exploration of these microorganisms could very well contribute to a deeper understanding of evolution and uncover some missing branches in the tree of life [3].

Culture-dependent and culture-independent methods have both been used to analyze the microbial composition of thermal springs. Culture-dependent methods have several limitations, the main one being that less than 10% of the total number of microorganisms in the environment are estimated to be routinely cultivable [8]. This limitation is primarily due to differences between laboratory and environmental conditions, the latter being often difficult to mimic [9]. However, cultivating microorganisms in pure cultures is crucial for describing their bioactive potential as well as for assigning function to genes previously identified by culture-independent methods, thus bridging the gap between culturing and culture-independent methods [10]. On the other hand, the large amount of sequencing data obtained by culture-independent methods enables the description of a much more comprehensive range of microorganisms than is possible using traditional culture-dependent approaches. Sequencing the ubiquitous prokaryotic 16S rRNA marker gene has been a cornerstone of culture-independent microbial community analyses for decades [11–13]. The major limitation of 16S rRNA gene sequence analysis is that it does not provide functional information that can be determined using metagenomic or pure culture analyses. Thus, a combination of marker gene sequence analyses and culture-dependent methods have been successfully used to analyze microbial diversity in many oligotrophic and extreme environments, including thermal springs [14–16].

Underground water systems are ubiquitous and can serve as an insight into the subsurface microscopic world [17]. Their investigation provides an opportunity to uncover unique prokaryotic taxa in many places on Earth [18–22]. Amongst the most renowned localities are the hot springs of Yellowstone National Park, where culture-independent analyses have discovered numerous hitherto uncultured microorganisms, mostly novel clades of Archaea [23]. Other widely investigated thermal springs in volcanic zones of New Zealand have been found to harbor unique microbial communities conditioned by temperature, pH, and geographical location [18, 24]. The warmest Czech thermal springs are located in the renowned spa town of Karlovy Vary (Carlsbad). This town is located where the Eger Rift meets the Karlovy Vary Thermal Spring Line, separating the Saxothuringian and Teplá-Barrandian tectonostratigraphic zones in the Bohemian Massif [25]. This intercontinental rift was created during the Neogene tectogenesis, with thermal springs being estimated to have first emerged in the area around 230,000 years ago. Their genesis was allowed thanks to the combination of terraced bedrock and alkaline magmatism [26]. This combination led to volcanic activity which caused a release of CO_2_ and results in bicarbonate-sulfate–chloride-enriched waters containing gaseous and dissolved CO_2_ [27]. Due to their chemical composition, these waters are used for gastrointestinal tract therapies and other balneological procedures. The balneological attraction of this town began as early as the fourteenth century and has continued to grow since, with more than a million visitors to its spas recorded in 2019 (https://www.karlovyvary.cz). Despite their extensive use and recognition as a natural and cultural heritage site, only a few studies have been conducted on the microbial communities colonizing these thermal springs [28–30]. The first study by Pěčková et al. (1991) used a culture-dependent approach and was exclusively focused on one bacterial genus, *Thermus* [28]. The other two studies [29, 30] focused on the analysis of specific biomolecules from a few selected isolates. However, a complete picture of the microbial life inhabiting the Karlovy Vary thermal springs has remained missing thus far.

In this study, we provide insights into the composition of prokaryotic communities in four Karlovy Vary thermal springs. Phylogenetic analysis of 16S rRNA gene sequencing data highlights the unique composition of prokaryotic communities in each spring and reveals the presence of as-yet-undetected taxa that form a major part of the microbial communities therein. We also characterize a collection of bacterial isolates, including some novel species, which were obtained using different culturing media. To reveal the potential of the isolates for biotechnological applications, we further investigate their tolerance to NaCl and higher temperatures by observing their growth under these types of stress. Finally, a whole-genome analysis of the phylogenetically novel bacterial species is provided.

## Methods

### Sampling of thermal spring water

Four thermal springs differing in their chemical composition and temperature (Additional file 1: Table S1) were examined in this study, Vřídlo (V, 72.0 °C), Mlýnský (M, 59.3 °C), Sadový starý (S, 46.3 °C), and Štěpánka (P, 18.3 °C). Samples were collected at two time points, in autumn 2018 and spring 2019. A total volume of 25 L of water was collected directly from the constructed pipes into sterile 2 L and 1 L glass bottles (SIMAX, CZ). To control for the asepticity of the collection process, the fallout of possible air contamination was sampled using the same type of 2 L sampling bottle filled with sterile deionized water, which was left open during the whole process of sampling. Collected samples and control bottles were immediately transferred to the laboratory (< 4 h). Cells were filtered onto 0.22 µm membrane filters (VWR, USA), and membrane filters with retained cells from 20 L of water were used for the extraction of metagenomic DNA, whereas filters with retained cells from the 3 L of water were used for the cultivation of microorganisms. The remaining 2 L were used for the enriched cultivation approach.

### Metagenomic DNA extraction

Metagenomic DNA was extracted from the filters using a DNeasy PowerWater Kit (QIAGEN, DE) according to the manufacturer's instructions. DNA concentrations were measured with a Qubit fluorometer using a dsDNA high-sensitivity assay (Invitrogen, USA) and extracted DNA was used for 16S rRNA gene sequencing.

### Amplicon sequencing of 16S rRNA genes

Primers targeting the variable V4–V5 region of the 16S rRNA gene, 515F 5′-GTGYCAGCMGCNGCGG-3′ and 926R 5′-CCGYCAATTYMTTTRAGTTT-3′ (Fraraccio et al*.* [31] adapted from Parada et al*.*[32] and modified according to Klindworth et al*.* [33]), were used for the PCR. The final PCR volume was 15 µL and contained 1 × KAPA HiFi HotStart ReadyMix (Kapa Biosystems, USA), 0.3 µM of each primer, and extracted metagenomic DNA (< 10 ng). The amplification was performed for 5 min of initial denaturation at 95 °C; followed by 25 cycles of: 20 s of denaturation at 98 °C, 15 s of annealing at 50 °C, and 40 s of elongation at 72 °C; and ended with final elongation for 5 min at 72 °C. Amplicons (0.5 µL) were used as templates for a second round of amplification using primers with sequencing adapters as described previously [31]. PCR was performed as described above except that the total volume was increased to 25 µL, primers were increased to 1 µM each, and the number of cycles was decreased to 10. The amplicons tagged with adapters were purified using AMPure XP Beads (Beckman Coulter, USA; bead:DNA ratio 0.8:1). Further library preparation and sequencing were performed in the DNA Core Lab of the University of Alaska Fairbanks, USA as follows: the concentration of amplicons was normalized to 1–2 ng using a SequalPrep Kit (Thermo Fisher Scientific, USA), the samples were pooled, subjected to 8-cycle PCR to add sequencing adapters, and sequenced in an Illumina MiSeq using the MiSeq Reagent Kit v3. In summary, 40 DNA samples were sequenced, including technical duplicates of: (1) each spring sample collected at two time points, (2) 4 samples of airborne contaminant control, (3) 5 blank samples, and (4) 3 mock community samples.

### Sequencing data processing

Obtained sequencing data were processed in the R environment [34]. The steps of the DADA2 pipeline [35] were followed with the following exceptions: maximum expected error was set to 1 (maxEE = 1) and truncation length set to 250 for the forward fastq files. Taxonomy was assigned to unique amplicon sequence variants (ASVs) using the SILVA SSU r138 database [36]. A mock community of 15 bacterial strains (Additional file 1: Table S2) was analyzed to ensure sequencing accuracy. The package phyloseq [37] was used for data handling and visualization. All MiSeq reads were deposited in the NCBI Short Read Archive under SRA study number PRJNA781448.

Five replicates of blank samples were included as controls for the entire process from DNA to amplicons and their processing. The R package Decontam [38] was used for detection and removal of contaminating ASVs, using both frequency-based and prevalence-based methods. Moreover, both eukaryotic ASVs and ASVs present in aseptic sampling controls were removed manually from the dataset. Sequences identified as contamination accounted for < 0.05% of all reads. Sequencing coverage was calculated using the package iNEXT [39].

Phylogenetic novelty at the taxonomic rank class was confirmed using the script SSUnique with default settings [40]. Novel ASVs were aligned using SINA aligner [41] with the five closest references from the prokaryotic tree of life based on 16S rRNA gene. Constructed phylogenetic tree was visualized using the package ggtree [42] in R.

### Microbial culturing

Two cultivation approaches were used for the isolation of indigenous microorganisms from the thermal spring waters. In the first approach, further referred to as cultivation with concentrated inoculum, cells retained from 2 L of spring water were resuspended overnight in 25 mL of filtered water from each respective spring. One hundred µL of the resulting cell suspensions was used to inoculate seven different oligotrophic media in 6 replicates (Additional file 1: Table S3). Distilled water in the media was replaced with filtrates from the corresponding thermal spring [43], and the plates were solidified using Noble Agar (1.8%). The plates were incubated at 58 °C (samples V, M, P), 44 °C (S, M, P), and 16 °C (P) for over 2 months. Cultivation temperatures of thermal spring P were selected to correspond to both those underground and at the outflow (Additional file 1: Table S1).

In the second approach, further referred to as cultivation with enriched inoculum, 2 L of unfiltered water from each thermal spring was incubated in sterile bottles for over 2 months in the dark at 37 °C. After that, 200 µL of water was inoculated into different concentrations of Luria–Bertani liquid medium (1/1, 1/2, 1/5, 1/10, 1/20, and 1/50 medium LB). After 1 week of cultivation in liquid media at 37 °C and 130 rpm, the enriched cultures were plated on diluted solid media (Plate Count Agar) in 5 replicates for each concentration of LB medium, and incubation continued at 37 °C.

During both cultivation approaches, solid media were screened for the appearance of bacterial colonies, which were continuously re-streaked. The resulting isolates were subjected to mass spectrometric analysis as described earlier by Strejcek et al*.* [44].

### Characterization of microbial isolates

The obtained collection of microbial cultures was identified using MALDI Biotyper® 3.1 (Bruker Daltonics, Germany) and dereplicated based on the similarity of protein mass spectra as described earlier [44]. Briefly, obtained spectra were clustered using a 0.9 cosine similarity cut-off; a representative isolate from each cluster was selected for further taxonomic identification either by mass spectrometry analysis [44] or 16S rRNA gene Sanger sequencing. Dereplicated isolates were tested for salt tolerance and lethal temperature. Salt tolerance in the range of NaCl 0–15_w/v_% was monitored by a single serial dilution spotting method adapted from Thomas et al. [45]. In brief, two technical replicates of bacterial cultures, standardized to 0.5 McFarland, were serially diluted three times and 20 µL of each dilution was spotted on solid R2A plates containing varying amounts of salt. The number of colony-forming units (CFUs) was recorded after 72 h of incubation. The lethal temperature of isolates was determined by heating standardized inoculum for 11 min (1 min of temperation, 10 min of incubation) to the examined temperature. Subsequently, the inoculum was streaked on R2A solid media and the viability of isolates was confirmed by the growth of colonies after 72 h. The range of examined lethal temperatures was 50–100 °C with a 5 °C increment. Both characterizations were performed in two biological replicas. The R packages ggplot2 [46], DECIPHER [47], and ape [48] were used for the further visualization and processing of cultivation data.

### Genome sequencing of phylogenetically novel isolates

To isolate genomic DNA of phylogenetically novel isolates, cultures were grown overnight in liquid LB media. Genomic DNA was isolated from cell pellets with a PureLink™ Genomic DNA Mini Kit (Invitrogen™, USA) following manufacturer’s instructions. The resulting DNA was subjected to library preparation and sequencing using a Nanopore MinION instrument (FLO-MIN106 flow cell) by following the same protocols as described in Lopez-Echartea et al*.* [49]. Base-calling was performed by using guppy (3.2.10) [50], adapters were trimmed using Porechop (https://github.com/rrwick/Porechop) and reads were quality filtered using Filtlong (https://github.com/rrwick/Filtlong). Sequencing statistics for all novel genomes are provided in Additional file 1: Table S6. Genome assembly was carried out using Canu software (v1.7) [51]. The obtained genome assemblies were polished using Medaka (v1.4.3) (https://github.com/nanoporetech/medaka), which maps the Nanopore reads to the draft assembly. The quality of the genome assemblies was evaluated with CheckM [52], available on the Kbase server [53].

Genomes were annotated using Prokka [54] and the PATRIC online server [55]. Additionally, KEGG Decoder was used to evaluate the metabolic potential of the strains [56]. The 16S rRNA gene sequences were searched against the EZtaxon database. Taxonomic affiliations of potentially novel strains were deduced by retrieving genomes of the most closely related taxa and subjecting them to OrthoANI [57] and EzAAI analyses [58]. Phylogenetic relationship of each strain to its most closely related taxon was established by computing the core gene phylogenetic trees using UBCG with default settings [59]. Constructed trees were visualized using MEGAX [60]. Genomic dissimilarities between two strains belonging to the same genus were derived by employing BPGA [61]. The obtained unique genes from each strain were identified by using KofamKOALA [62] and were graphically interpreted using STAMP (v2.1.3) [63]. All genomic data generated in this study are available under the NCBI BioProject ID PRJNA772595.

## Results

### Amplicon sequencing of 16S rRNA genes

A total of 1,442,275 16S rRNA gene sequence reads were retrieved after DADA2 pipeline processing, averaging 180,284 sequences per sample, with a minimum of 66,149 sequences per sample. Of those, 2,430 unique ASVs were identified. While determining sequencing accuracy based on mock community analysis, we found that 98.33% ± 0.25 pp of reads were 100% identical to those expected.

**Fig. 1:**
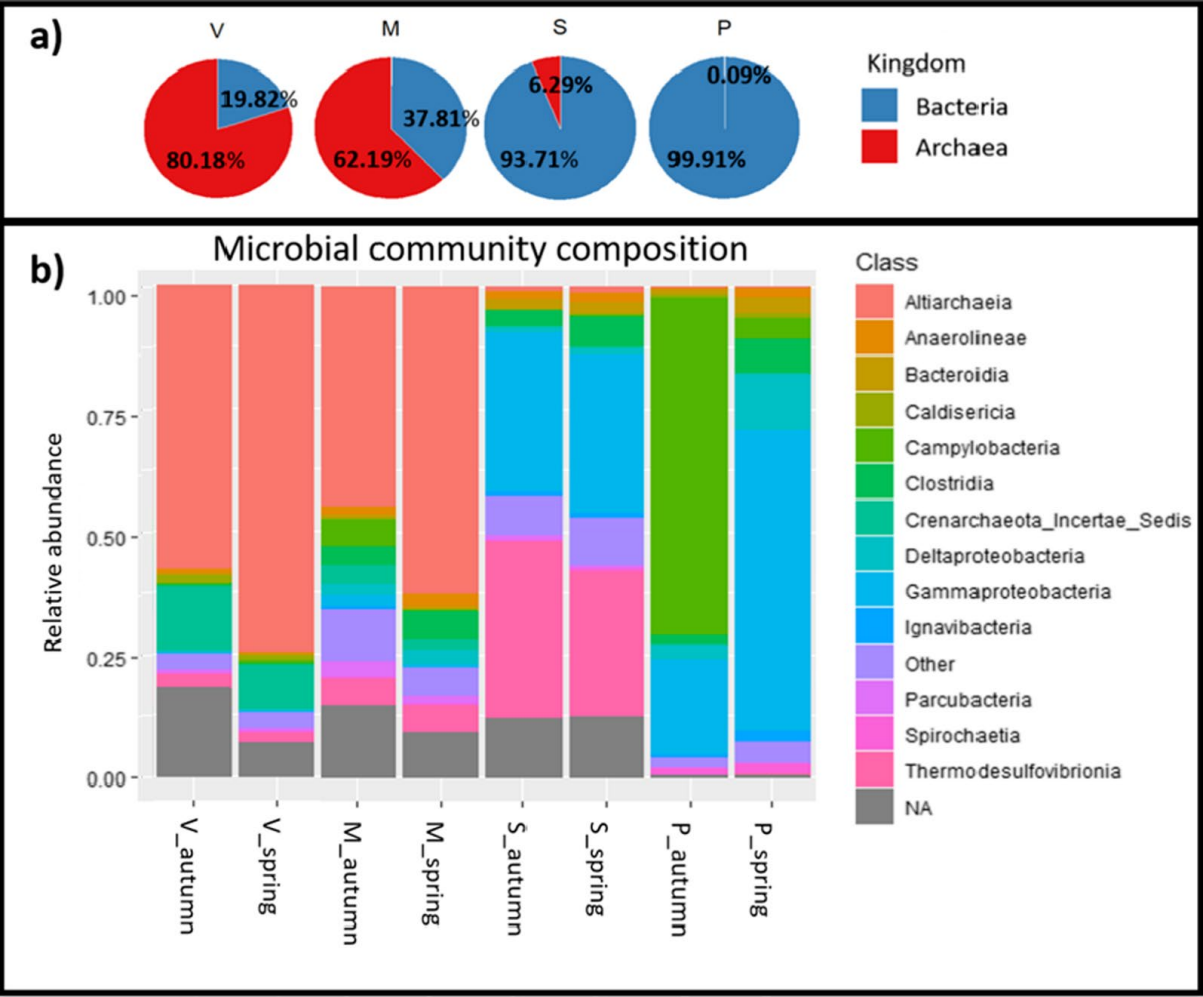
Microbial community composition of the four examined Karlovy Vary thermal springs. Pie charts **a** show the fractions of ASVs belonging to Bacteria or Archaea (counted from combined spring and autumn sequencing data). Bar plots **b** show the community composition at the taxonomic level of class for each thermal spring at the two sampled time points (autumn and spring). Classes referred to as other represent all classes with a relative abundance < 0.05% per sample; NA refers to unclassified ASVs

**Fig. 2:**
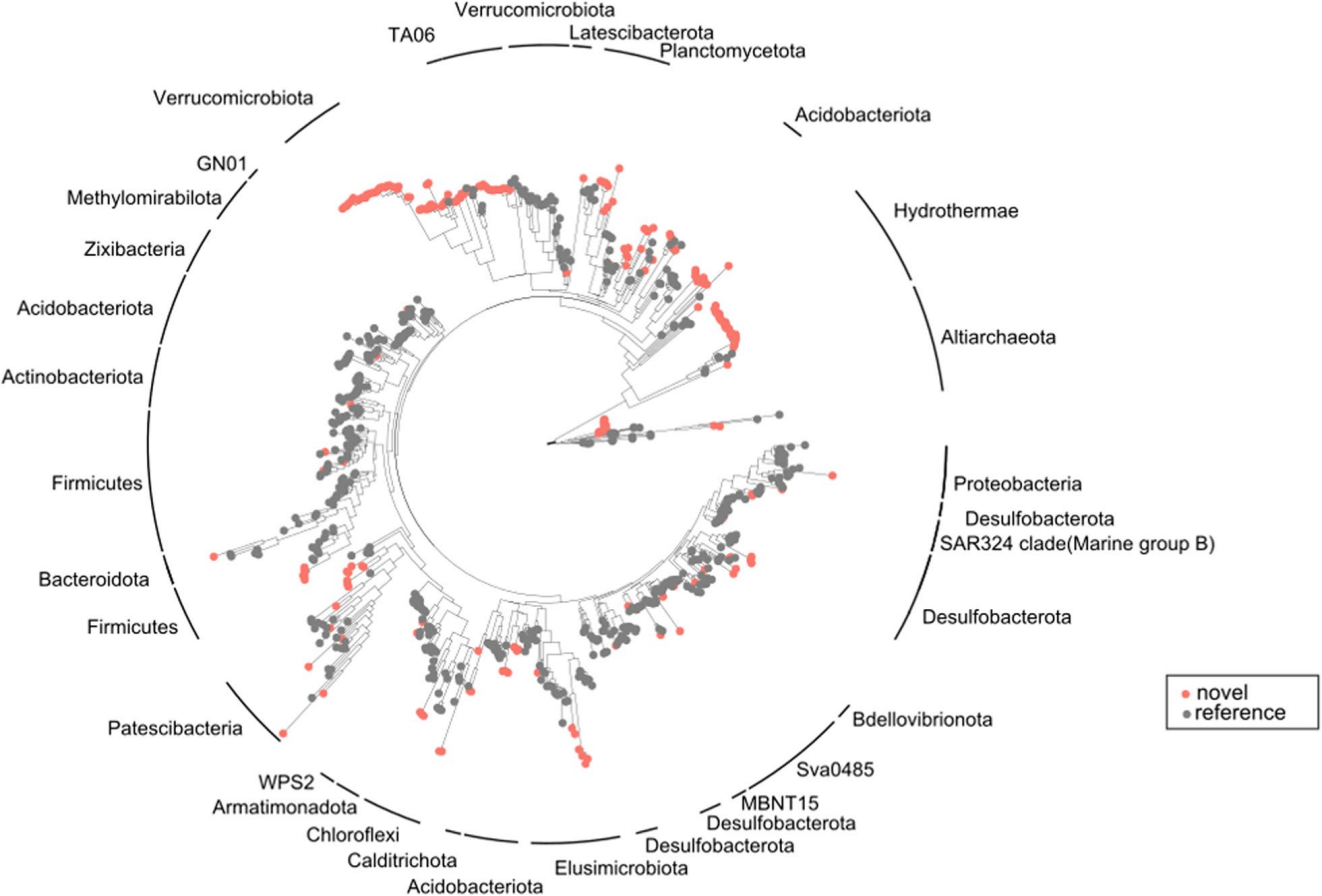
Phylogenetically novel ASVs (at the taxonomic rank of class) positioned in the prokaryotic tree of life. Red dots: novel ASVs detected in this study; grey dots: closest references of novel ASVs

The microbial composition significantly differed in the sampled springs (permutation test for CCA, R^2^ = 0.7484, *p *value = 0.007), with the proportion of archaeal ASVs increasing with increasing water temperature (Fig. 1a). In total, 68% of ASVs detected remained unclassified at the genus level. In the warmest spring, Vřídlo, unclassified ASVs comprised 97% of all reads (Additional file 1: Table S4). However, members of several well-described genera were observed amongst the 50 most abundant unique ASVs from each spring. These genera included the chemoheterotrophic thermophilic genera *Thermoflexus* and *Hydrogenobacter,* the facultatively anaerobic genus *Anaerobacillus* in the warmest spring V. Genera involved in the metabolism of nitrogen, such as *Candidatus* Nitrotoga (S, P), as well as iron and sulfur, such as *Rhodoferax* (P), *Sideroxydans* (S), *Desulfacinum* (P), *Thiomonas* (S), *Thermodesulfitimonas* (P), *Halothiobacillus* (P), *Sulfurimonas* (M, P), *Sulfuricurvum* (P), and *Thermoanaerobaculum* (M). The genera *Rhodoferax* and *Sulfurimonas* represented over 60% of the total reads in the coolest spring (P), with *Sulfurimonas* being predominant in the autumn, and *Rhodoferax* in the spring. Such inconsistency in microbial composition between the autumn and spring was only observed in thermal spring P. Despite this inconsistency, all examined thermal springs did not significantly differ in their seasonal microbial community composition (Pairwise multilevel comparison using adonis, R2 = 0.046, *p* value = 0.125). The number of potentially novel classes of prokaryotes that could not be classified using the SILVA_SSU_r138 database increased with increasing temperature of the thermal spring (Fig. 1b). While for the coolest spring P < 2% of reads were unclassified at the level of class, this number was 17% in springs S and M, and 19% in the warmest spring V. These reads represent potentially novel microorganisms and accounted for 226 novel bacterial ASVs and 60 archaeal ASVs (Fig. 2). In fact, several entirely novel clades of ASVs were detected across the phylogenetic tree, the largest of which were in the phyla Verrucomicrobiota, TA06, Hydrothermae (Bacteria), and Altiarchaeota (Archaea) (Fig. 2).

### Cultivation with concentrated and enriched inocula

**Fig. 3:**
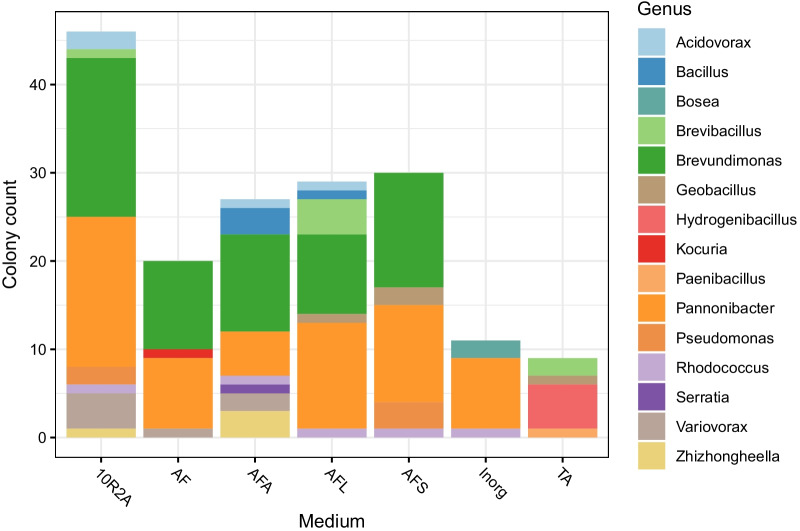
The number and distribution of microorganisms isolated on different oligotrophic media during cultivation with concentrated inocula. Acronyms for media: 10R2A, 10 × diluted Reasoner's 2A medium with filtrate of corresponding thermal water instead of distilled water; AF, Noble agar and filtrate; AFA, Noble agar, acetate and filtrate; AFL, Noble agar, lactate and filtrate; AFS, Noble agar, succinate and filtrate; Inorg, inorganic medium; TA, thermus agar

**Fig. 4:**
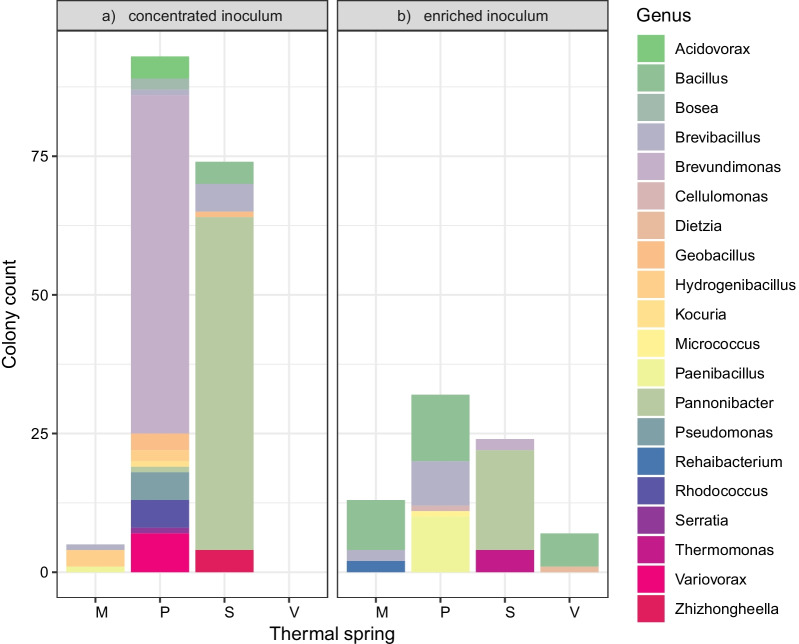
Microbial cultures isolated from different thermal springs using cultivation with concentrated inoculum (**a**) and cultivation with enriched inoculum (**b**)

The concentrated inoculum cultivation approach with thermal water resulted in the isolation of 172 isolates before dereplication, with the distribution and yield differing based on the oligotrophic medium used (Fig. 3). The cultivation on 10 × diluted R2A agar yielded the highest number of isolates. Other media with filtered spring water yielded similar numbers of isolates, while the specialized media for autotrophs (inorganic medium) and the genus *Thermus* (thermus agar) yielded the lowest number of isolates, eleven and nine, respectively. Despite the lower number of isolates grown on these media, they provided unique cultures of the genus *Hydrogenibacillus* and *Bosea*. None of the isolates originated from thermal spring V, and members of only three genera, *Brevibacillus*, *Hydrogenibacillus* and *Paenibacillus* were isolated from thermal spring M, which was the second warmest spring. The two other thermal springs, S and P, provided more diverse isolates, although they were both dominated by one major genus, *Pannonibacter* and *Brevundimonas*, respectively (Fig. 4a). The enriched inoculum cultivation approach resulted in 76 isolates before dereplication. In contrast to the concentrated inoculum approach, seven isolates were isolated from the warmest sample V, which were identified as *Bacillus* and *Dietzia*. Furthermore, members of the genera *Rehaibacterium* (thermal spring M), *Thermomonas* (thermal spring S), *Paenibacillus* and *Cellulomonas* (thermal spring P) were isolated. Similarly to the cultivation with concentrated inoculum, the genus *Pannonibacter* dominated the isolates from thermal spring S (Fig. 4b). All isolated strains were bacteria and were affiliated with the phyla Actinobacteria, Firmicutes, and Proteobacteria.

### Characteristics of the cultures

**Fig. 5:**
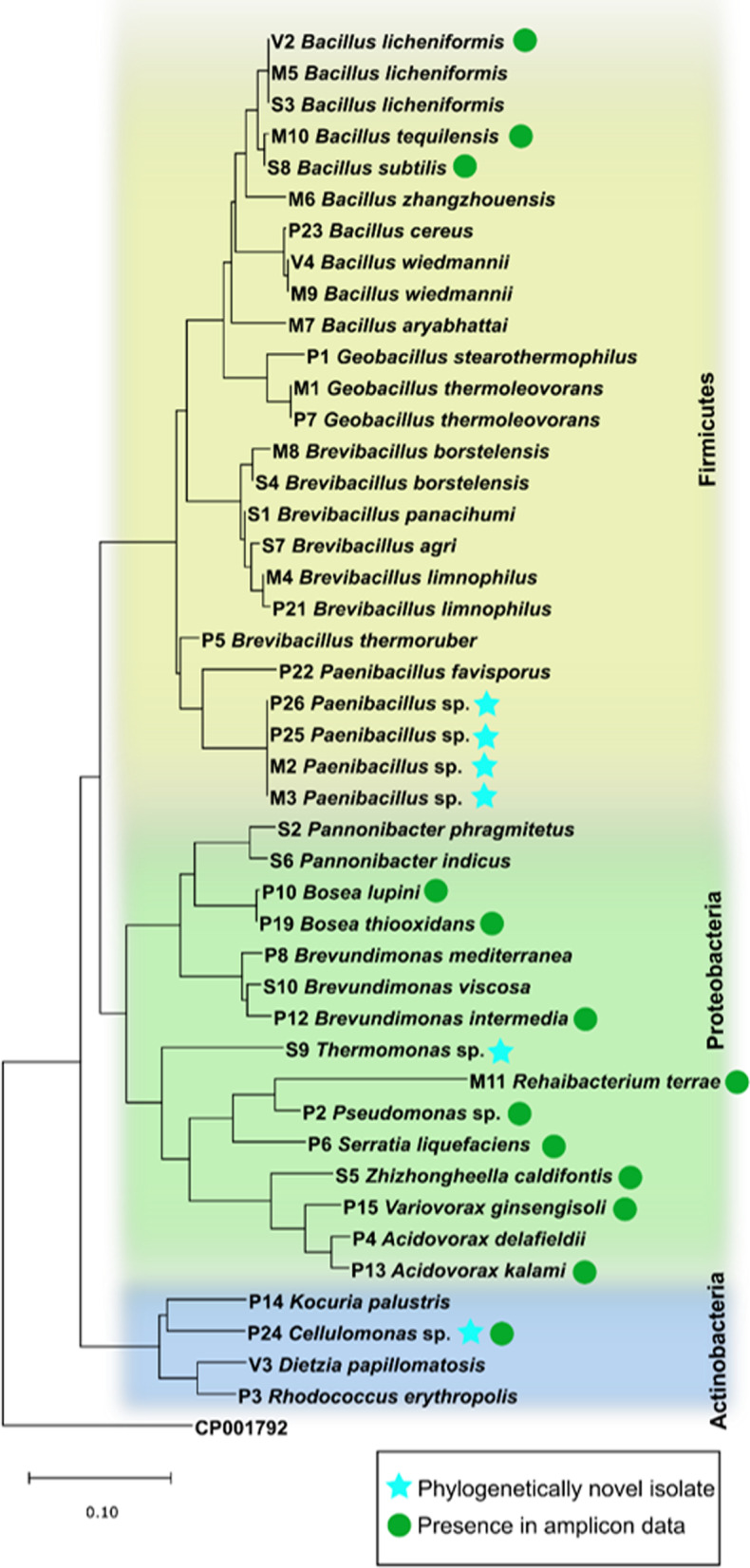
Phylogenetic tree based on 16S rRNA gene sequences of unique thermal spring isolates. The neighbor-joining phylogenetic tree was constructed using the distance model JC69. The outgroup sequence with accession number CP001792 represents *Fibrobacter succinogenes* subsp. *Succinogenes* S85. The green dots represent a match of the 16S rRNA sequence of the culture to ASVs. The blue stars represent potentially novel species

Mass spectrometry-based dereplication of the collection of 248 isolates from both cultivation approaches resulted in the identification of 44 unique taxa (Fig. 5). The 16S rRNA gene sequence analysis of this dereplicated collection identified six potentially novel species. Isolates M2, M3, P25, and P26 were identified as members of the genus *Paenibacillus*. These four isolates had identical 16S rRNA gene sequences, but were not dereplicated due to their different origins (spring M and P) and morphologies (M2 and P25 form whitish colonies on solid media, whereas M3 and P26 form transparent colonies). The remaining two isolates, S9 and P24, were identified as members of the genera *Thermomonas* and *Cellulomonas*, respectively.

The 16S rRNA gene sequences of the dereplicated collection of microbial isolates were compared to ASVs in the amplicon data set in order to estimate the abundance of the culturable microorganisms in the springs. Thirteen sequences from the isolates were identical to sequences in the amplicon data. The remaining 30 isolate sequences did not match any ASVs despite the sequencing effort, which indicated that sample coverage was over 99% for each sample (Additional file 1: Table S5). These 30 isolate sequences mostly belonged to the order Bacillales, which is known for its ability to form endospores [64].

**Fig. 6:**
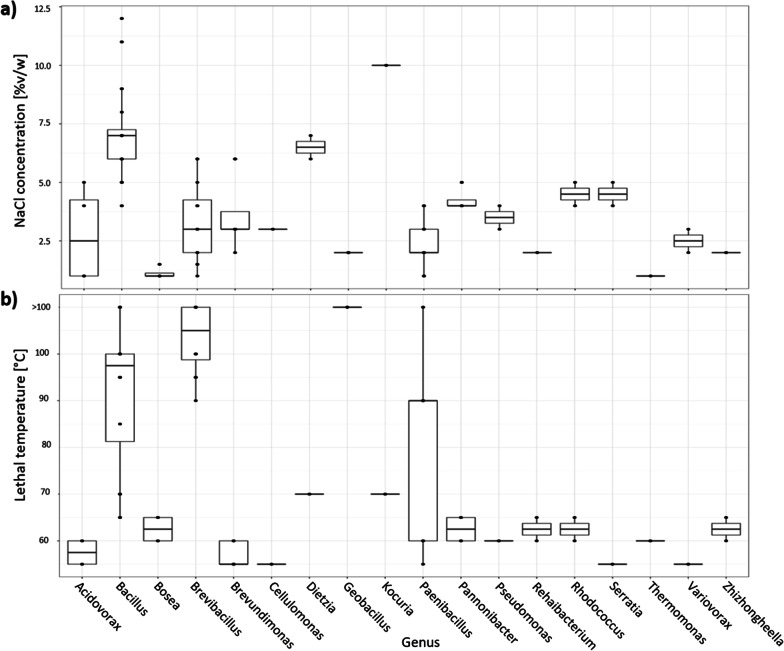
Characterization of isolated microorganisms for salt tolerance (**a**) and lethal temperature (**b**). Data points can represent one or more values since the tests were performed in duplicates

The collection of isolates was characterized in terms of salt tolerance and lethal temperature (Fig. 6). Over 60% of the isolates were able to grow at > 3% NaCl, which is approximately the salt concentration of seawater [65] (Fig. 6a). Over 25% of the isolates were able to grow at ≥ 6% NaCl and can be considered halotolerant [66]. The most salt-tolerant isolate was *Bacillus zhangzhouensis* (isolate M6), which was able to grow at 12% NaCl. Overall, the majority of *Bacillus* isolates were able to grow at > 5% NaCl. Other halotolerant genera included *Kocuria* (isolate P14, 10% NaCl) and *Dietzia* (isolate V3, 7% NaCl).

The lethal temperature of the majority of isolates was between 55 and 70 °C (Fig. 6b). However, the isolates of *Brevibacillus* and some isolates of the *Bacillus* and *Paenibacillus* genera were able to survive temperatures > 80 °C. Notably, the lethal temperature of isolates M6, M9, M10, M12, and S1 was 100 °C; moreover, isolates M1, M4, M8, S3, P7, P21, and P22 were able to survive exposure to boiling water, thus their lethal temperature is presented as > 100 °C. Overall, trends in salt and temperature tolerance were related to bacterial genera rather than the isolation source. Spearman's rank correlation between salt and heat tolerance was 0.273 (*p* value = 0.009).

### Genomic analysis of phylogenetically novel isolates

The four isolates *Thermomonas* sp. S9, *Paenibacillus* sp. P25, *Paenibacillus* sp. P26 and *Cellulomonas* sp. P24 were found to have 16S rRNA gene sequence identities of < 98.65% to their closest cultured relatives, indicating their potential for representing phylogenetically novel species. Therefore, their full genomes were sequenced and analyzed. Genome statistics, including the number of scaffolds, total size, G + C content, number of coding DNA sequences (CDS) and protein-coding sequences, completeness and contamination of these isolates are given in Additional file 1: Table S6. The phylogenetic trees based on whole genome marker genes are provided in the Additional file 1: Figs. S8–S10.

### *Thermomonas* sp. S9

**Fig. 7:**
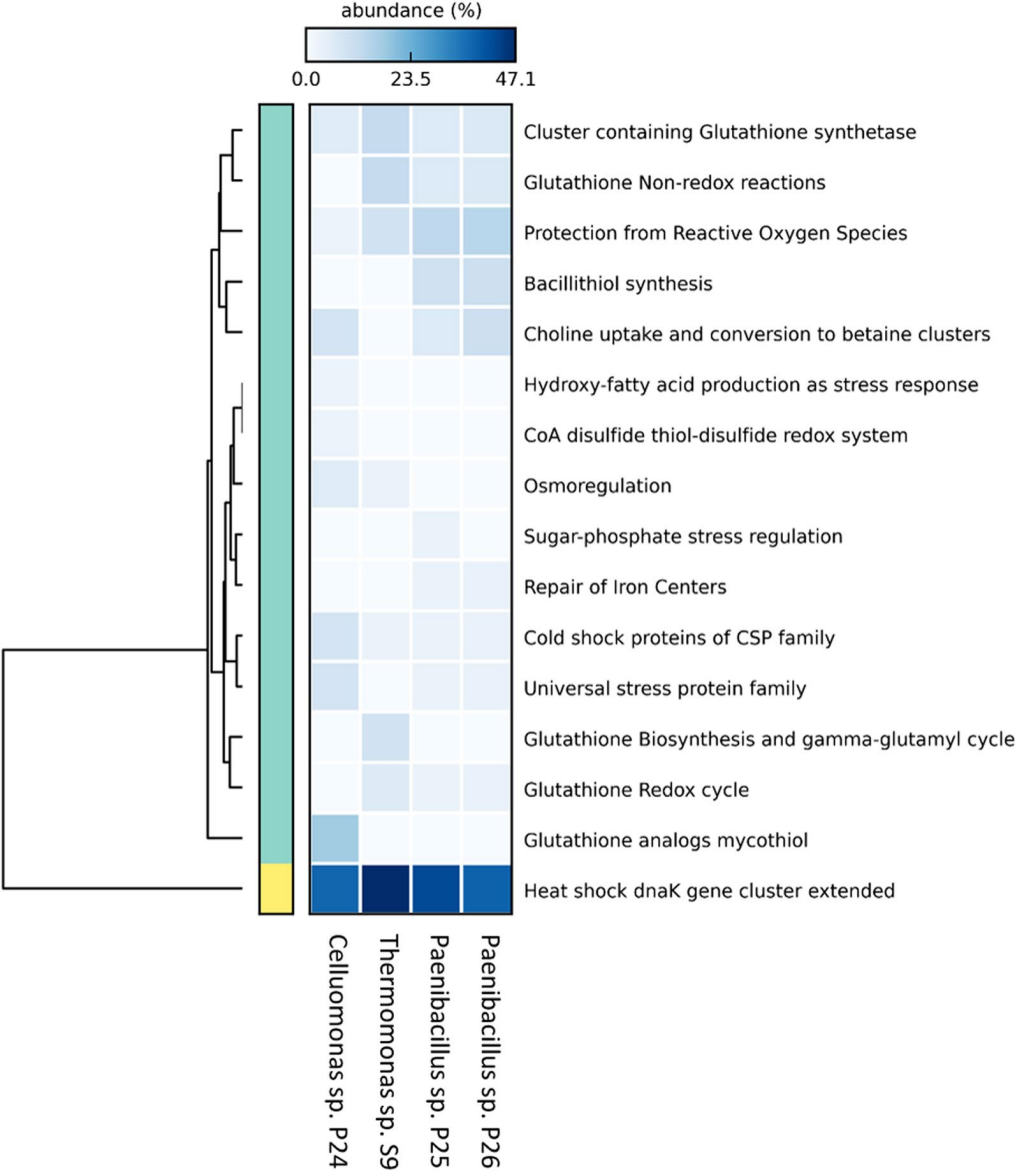
Prevalence of stress-response genes detected in phylogenetically novel taxa from the Karlovy Vary thermal springs. The scale of the heatmap denotes the gene copy number (represented as relative abundance), and the dendrogram depicts the UPGMA-based clustering of genes

The strain S9 exhibited 98.5% 16S rRNA gene sequence similarity to *Thermomonas haemolytica* A50-7-3^T^. OrthoANI values for the S9 genome and available genomes of the related members of the family *Lysobacteraceae* were in the range of 92.8–81.4% [67]. The results of core-gene-based phylogeny depicted the monophyletic clustering of strain S9, showing divergence from the type genera, such as *Thermomonas*, *Luteimonas,* and *Pseudoxanthomonas*. The distribution of functional categories revealed the predominance of genes belonging to metabolism, energy, protein processing, cellular processes, stress response, defense, virulence, and membrane transport (Additional file 1: Table S7). Strain S9 was found to harbor a wide range of stress and heat response genes of which up to 50% belong to the heat shock *dnaK* gene cluster. Furthermore, the strain S9 was found to harbor genes for glutathione biosynthesis and gamma-glutamyl cycle, which were absent in all other strains (Fig. 7). Collective observations of the genomic characteristics provide evidence that strain S9 represents a novel species of the genus *Thermomonas* in the family Lysobacteraceae, phylum Proteobacteria.

### *Paenibacillus* sp. P25 and *Paenibacillus* sp. P26

The strains P25 and P26 showed 96.8% 16S rRNA gene sequence identity to *Paenibacillus konkukensis* SK-3146^T^. An OrthoANI comparison between the available genomes of the genus *Paenibacillus* and the genomes of the P25 and P26 strains resulted in values of ≤ 79.5%. OrthoANI homology of strains P25 and P26 was 99.7%, and average amino acid identity was 99.1%. Additionally, core-gene-based phylogenic analysis revealed a separate clustering of P25 and P26 with the *Paenibacillus* genus (Additional file 1: Fig. S9). Genome annotations of the two novel strains revealed subtle differences in genomic architecture. Both strains featured various gene clusters for stress response, most of which were shared, except for sugar-phosphate stress regulation, which was only featured in strain P25 (Fig. 7). Based on the genomic characterization, we propose strains P25 and P26 to represent members of a novel species of the genus *Paenibacillus* in the family Paenibacillaceae, phylum Firmicutes.

Although strains P25 and P26 exhibited a high degree of sequence similarity (Fig. 7), some phenotypic differences were detected. Whereas strain P25 can grow at 3% NaCl and its lethal temperature is 90 °C, strain P26 can grow at 2% NaCl and its lethal temperature is 60 °C (Fig. 6). These differences could potentially be explained by the observed subtle divergence in their genomes. A genome-genome comparison revealed the presence of 49 and 36 unique genes for strains P25 and P26, respectively, as well as differences in the abundances of KEGG categories (Additional file 1: Fig. S11). The strains P25 and P26 encoded for, respectively, 76 and 77 antibiotic resistance genes, including vancomycin resistance, as detected by PATRIC (Additional file 1: Table S6).

### *Cellulomonas* sp. P24

The strain P24 showed 97.9% 16S rRNA gene sequence similarity with *Cellulomonas cellasea* DSM 20118^T^. OrthoANI comparisons between related members of the *Cellulomonadaceae* family and strain P24 was ≤ 79.8%. Additionally, the results of core-gene-based phylogeny depicted monophyletic clustering of strain P24, exhibiting divergence from the type species of the genus *Cellulomonas*. The distribution of functional categories included metabolism, energy, protein processing, stress response, defense, virulence, cellular processes, and finally membrane transport. The genome was shown to also feature a CRISPR array. The strain P24 was found to harbor the glutathione analog mycothiol, a CoA disulfide thiol-disulfide redox system, and hydroxy-fatty acid production genes as unique functional traits which were absent in the other genomes (Fig. 7), The results from genomic characterization and phylogenetic assessment indicate that strain P24 is a novel species of the genus *Cellulomonas* in the family Cellulomonadaceae, phylum Actinobacteria.

Despite originating from the same thermal spring area, all novel strains harbored notably distinct gene complexes, which are presumably required to cope with stress in their extreme environmental niches (Fig. 7).

## Discussion

### Microbial community composition

In our study, we used a combination of 16S rRNA gene amplicon sequencing analysis and microbial cultivation approaches to analyze the microbial community composition of four Karlovy Vary thermal springs differing in their temperature and chemical composition. Figure 1 depicts the presence of up to 19% as-yet-unclassified, potentially novel microbial taxa at the class level. Despite this, the set of 50 most abundant unique ASVs from each spring contains genera that are commonly reported in literature on similar aquatic habitats, such as the genera *Desulfacinum* and *Thiomonas* [68], *Sulfuricurvum* [69], *Thermoflexus* [70], *Hydrogenobacter* [71], *Sideroxydans* [72], *Halothiobacillus*, or *Sulfurimonas* [73], some of which in similar relative abundances compared to our dataset. Genera known to be involved in sulfate reduction [74] and iron oxidation [75] were found mainly in thermal spring P, which is also the spring with the highest iron and sulfate cation concentrations of the examined thermal springs (Additional file 1: Table S1).

### Microbial cultivation

The two cultivation approaches yielded a collection of 44 unique bacterial isolates. The lowest number of obtained colonies originated from thermal springs V and M (Fig. 4), which can be ascribed to the high abundance of archaea, whose cultivation is generally very difficult [76, 77], and previously undetected taxa (Fig. 1). The very low rate of culturability may have also been connected with the presence of VBNC microorganisms that use the uncultivable state as a response to environmental stress such as high temperature [78].

Although the concentrated inoculum approach yielded a higher number of isolates (Fig. 4a), the enriched inoculum approach led to the isolation of novel species belonging to rare taxa in our amplicon dataset (Fig. 4b), including novel species of genera *Thermomonas* (S), *Paenibacillus* and *Cellulomonas* (P). These findings confirm that such unconventional cultivation approaches are successful in isolating rare taxa [79] and that prolonged cultivation can enhance culturability of slow-growing and rare microbes [80]. At the same time, the enriched inoculum cultivation approach led to the enrichment of *Bacillus*-like genera, which are ubiquitous and often represent the cultivable part of microbes from thermal springs [15, 81]. Even though roughly a third of isolates were detected in the amplicon sequencing data, most of these taxa formed a very rare fraction of the amplicon dataset (0.019% of sequencing reads). These results are in line with the findings that the dominant taxa in the environment are often not recovered by microbial cultivation [82]. Most of the genera which were isolated but not found in the amplicon sequencing data belonged to either the genus *Bacillus* and related genera whose cells might have been present as spores in the environment and could be missed by sequencing analyses [83], or as Kurm et al*.* described in their study, represented cultivable rare taxa [84].

Extreme environmental conditions are challenging to mimic in the laboratory, further complicating the cultivation of microbes. However, alternatives to classical cultivation on Petri dishes exist. For example, some rare taxa can be enriched using dilution to extinction [85], an Ichip platform [86], or diffusion chambers simulating natural environments [87, 88]. Continued microbial cultivation efforts are crucial for the study of phylogenetically novel strains, as well as for the discovery of potential biotechnologically attractive strains and enzymes that are active under extreme conditions [89], such as the widely used Taq polymerase [90]. According to Raddadi et al. [7], thermozymes produced by thermophilic microorganisms are often able to resist proteolysis under a combination of high salinity and high temperature [91]. Thus, we characterized the collection of microorganisms in terms of both salt tolerance and tolerance to high temperatures (Fig. 6). We could not confirm a significant correlation between salt and high temperature tolerance (Spearman's rank correlation = 0.273, *p* value = 0.009). However, isolate M6 of the species *Bacillus zhangzhouensis* was found to be halophilic and able to tolerate extremely high temperatures (Fig. 6). Moreover, a strain of the same species previously demonstrated biotechnological application potential thanks to the production of alkaliphilic enzymes [92]. Even though this coupled resistance is highly likely to be associated with the ability of bacilli to form spores, the genus *Bacillus* and other members of the family *Bacillaceae* are commonly found to be enzymatically active in hot springs [93, 94]. In addition to the family *Bacillaceae*, the genus *Kocuria* exhibited tolerance to higher salt concentrations (Fig. 6a). It is in line with the previous findings on the tolerance of this genus to different harsh conditions and its biotechnological potential [95]. Further analyses of the collection of isolates for potential biotechnological use is beyond the scope of this study and will be a subject of future research.

### Phylogenetic novelty in thermal springs

Most of prokaryotic diversity likely remains unexplored [96], and so it is not surprising that an extreme environment, such as thermal springs, will host as-yet-undescribed taxa which may represent the majority of its microbial community. Such observations were made by Bourrain et al*.* [22] and Fasesan et al*.* [97] in recent years, with both groups pointing out that as-yet-undescribed taxa constitute a stable part of the microbial community in heavily used thermal waters. Bourrain’s group examined Avéne Thermal Spring Water (21 °C) and obtained 12% unassigned reads at the genus level, a number similar to what we obtained in the coolest spring, P (15%). In contrast, the number of unassigned reads at the genus level in the hottest spring V in our study comprised 98% of the total reads (Additional file 1: Table S4), a fraction that is commonly observed in hot springs [98]. Such a high representation of unassigned reads is caused by the presence of a potentially novel order of the class Altiarchaeia that dominates the two warmest springs, V and M (Fig. 1). The presence of novel archaeal taxa in hot springs is in agreement with previous studies, such as the one by Inskeep et al*.* [99], who were able to discover and describe a novel archaeal phylum in Yellowstone National Park’s geothermal springs by metagenomics.

Despite the high number of unassigned, thus potentially novel sequences in the amplicon data, there is often no confirmation or further analyses of these sequences. To avoid this, we used the tool SSUnique [40] to highlight phylogenetic novelty amongst the unassigned sequences in our amplicon dataset (Fig. 2). Four of the novel classes form extensive clades in the well-established phyla Hydrothermae, Altiarchaeota, Verrucomicrobia, and TA06. Phylum Hydrothermae was first identified by Jungbluth et al*.* in 2017 using high-quality metagenome-assembled genome analysis to describe the representative bacterium EM3 [100]. Since then, no members of this phylum have been cultivated, which is often the case in hot spring environments [101, 102]. In our dataset, putative members of this phylum were detected mostly in sample V, forming up to 14% of reads. Phylum Altiarchaeota was represented by 60 different ASVs that dominated samples V and M in our dataset (Fig. 2). Although this phylum is widespread in many aquatic habitats [103, 104], no members have been successfully cultivated yet. The first description was done by Probst et al*.,* who introduced *Candidatus* Altiarchaeum hamiconexum as a member of the phylum Euryarchaeota [103], although it was later reclassified as the novel phylum Altiarchaeota, which is a member of the superphylum DPANN. The members of Altiarchaeota are known to be autotrophs and biofilm-forming archaea, and thus represent a carbon sink in thermal springs [105]. Novel clades of the phyla Verrucomicrobia (M, P) and TA06 (S) formed a small fraction of our amplicon data, but their presence in various freshwater habitats such as acidic hot springs, thermal springs, or cold lakes has been repeatedly reported [106–110]. While no representatives of the phylum TA06 have been cultivated thus far, the phylum Verrucomicrobia has many characterized strains, although its ecological impact in aquatic habitats remains unclear [111]. To sum up, novel taxa, or entire clades of the aforementioned phyla, are great candidates for targeted genomics and could reveal important information about the ecology of thermal springs. Despite our cultivation efforts, we were unable to provide cultured representatives of novel clades of bacteria. To achieve this, live-FISH based on 16S rRNA genes combined with cell-sorting systems [112] could be used for single-cell genome sequencing and the genomic information can be later used for modeling the nutrient requirements [113] of sorted cells.

The modifications of common culture techniques used herein led to the isolation of several potentially novel species (Fig. 5). All the sequenced genomes of the four isolates had OrthoANI values < 95%, which is estimated as the cut-off for species demarcation [114], and are therefore presented herein as novel species (Fig. 5). Novel members of the genus *Paenibacillus* are introduced as two different strains of the same species. Based on the genomic information, both strains harbor antibiotic resistance genes, including resistance to vancomycin (Additional file 1: Table S6). Although this type of resistance was already found in the genomes of a *Paenibacillus* strain isolated from soil [115], it is not typically reported for members of this genus from environments with a lack of human impact, such as Karlovy Vary thermal springs. This specific resistance may play a role in the survival strategy for a stressful extreme environment with a low nutrient content [116, 117]. Due to the type of origin of the novel taxa, we focused our attention on the presence of stress response genes in their genomes. As expected, the most abundant stress response gene in our dataset was the heat shock *dnaK* gene cluster, associated with response to high temperatures (Fig. 7). *Thermomonas* sp. S9 harbored the highest abundance of these genes, corroborating with findings that this genus is often associated with warm to hot environments [118]. In fact, one of seven current members of the genus, the species *T. hydrothermalis*, has been commonly isolated from hot springs and used as a source of thermostable enzymes [119, 120]. In contrast, the genera *Cellulomonas* and *Paenibacillus* encompass many species and their appearance is almost ubiquitous, including thermal springs [121, 122]. Novel representatives of the genus *Paenibacillus* are very often found in thermal waters thanks to their ubiquity and ability to form spores [123–125].

## Conclusions

In summary, our study is the first to thoroughly analyze the microbial community composition of Karlovy Vary thermal springs. The modified cultivation approaches enabled us to obtain a collection of 44 indigenous microorganisms, including members of 3 novel species. Due to their extremophilic origins, these microorganisms can be further investigated for potential biotechnological applications. The taxonomic novelty of microbes inhabiting the Karlovy Vary thermal springs was further confirmed by 16S rRNA gene amplicon sequencing, revealing numerous classes of previously undescribed taxa. Future metagenomic analyses will further unravel the community structure and metabolic potential of Karlovy Vary thermal spring microbial communities. Such information will aid in defining subsequent targeted cultivation experiments that could lead to the discovery of novel extremophilic enzymes with the potential of decreasing water and energy consumption in industrial biotechnology.

## Supplementary Information

**Additional file 1.** **Table S1:** Chemical composition, temperature, and flow rates of the examined thermal springs. **Table S2:** Mock community composition. **Table S3:** List and composition of the used media. **Table S4:** Percentage of unclassified ASVs at different taxonomic levels. **Table S5:** Sequencing coverage computed using package iNEXT. **Table S6:** Genome statistics of the phylogenetically novel bacterial species and results from the annotation server PATRIC. **Table S7:** Functional categories distribution in the genomes of the phylogenetically novel bacterial species. **Figure S8:** UBCG-based core-genome phylogenetic analysis depicting the distinct positioning of strain S9 with members of the genus Thermomonas. Bootstrap values (expressed as percentages of 1000 replications) of above70% are shown at the branch points. **Figure S9:** UBCG-based core-genome phylogenetic analysis depicting the distinct positioning of strains P25 and P26 with members of the genus Paenibacillus. Bootstrap values (expressed as percentages of 1000 replications) of above 70% are shown at the branch points. **Figure S10:** UBCG based core-genome phylogenetic analysis depicting the distinct positioning of strain P24 with members of the genus Cellulomonas. Bootstrap values (expressed as percentages of 1000 replications) of above 70% are shown at the branch points. **Figure S11:** Differences in KEGG categories abundances in the genomes of strains P25 and P26.

## Data Availability

All MiSeq reads generated and analyzed during the current study are available in the NCBI Short Read Archive under SRA study number PRJNA781448. The genome data generated in this study are available under the NCBI BioProject ID PRJNA772595.
