# Supplementary material for: Prokaryotes of renowned Karlovy Vary (Carlsbad) thermal springs: phylogenetic and cultivation analysis
Source: Environ Microbiome. 2022 Sep 11;17:48. doi: 10.1186/s40793-022-00440-2 (PMC9465906; doi:10.1186/s40793-022-00440-2)

Additional file 1

Prokaryotes of renowned Karlovy Vary (Carlsbad) thermal springs: Phylogenetic and cultivation analysis

Tereza Smrhova[1], Kunal Jani[1], Petr Pajer[2], Gabriela Kapinusova[1], Tomas Vylita[3], Jachym Suman[1], Michal Strejcek[1], Ondrej Uhlik[1,✉]

[1]*University of Chemistry and Technology, Prague, Faculty of Food and Biochemical Technology, Department of Biochemistry and Microbiology, Prague, Czech Republic*

[2]*Military Health Institute, Ministry of Defence of the Czech Republic, Prague, Czech Republic*

[3] *Institute of Balneology and Spa Sciences, Karlovy Vary, Czech Republic*

✉**Corresponding author:** University of Chemistry and Technology, Prague, Technicka 3, 166 28 Prague 6, Czech Republic. Phone: +420 220 44 2020; email: ondrej.uhlik@vscht.cz

Keywords: amplicon sequencing analysis, cultivation analysis, thermal water springs, phylogenetic novelty

Table S1: Chemical composition, temperature, and flow rates of the examined thermal springs.

Sample	V	M	S	P
Temperature [°C]	72.0	59.3	46.3	18.3/60.2*
Flow rate [l.s^{-1}]	8.0	3	2.4	0.4
pH	6.86	6.67	6.56	6.36
Sodium (Na^{+}) [mg.l^{-1}]	1630	1650	1650	1790
Potasium (K^{+}) [mg.l^{-1}]	88.5	91.2	92.9	98.9
Calcium (Ca^{2+}) [mg.l^{-1}]	118	116	110	119
Magnesium (Mg^{2+}) [mg.l^{-1}]	42.4	43.4	40.2	41.7
Iron (Fe^{2+}) [mg.l^{-1}]	1.26	1.17	1.1	5.54
Amonium ion (NH_4^{+}) [mg.l^{-1}]	0.46	0.47	0.48	0.54
Cadmium (Cd^{2+}) [mg.l^{-1}]	0.00012	0.00004	0.0006	0.00002
Copper (Cu^{2+}) [mg.l^{-1}]	0.0049	0.00	<0.0003	0.00
Lead (Pb^{2+}) [mg.l^{-1}]	0.0037	0.0016	0.0001	0.0001
Bicarbonite (HCO_3^{-}) [mg.l^{-1}]	2150	2160	2150	2280
Chloride (Cl^{-}) [mg.l^{-1}]	595	609	604	659
Sulfate (So_4^{2-}) [mg.l^{-1}]	1630	1620	1610	1770
Nitrites (NO_2^{-}) [mg.l^{-1}]	<0.01	<0.01	<0.01	<0.01
Nitrates (NO_3^{-}) [mg.l^{-1}]	<0.50	<0.50	<0.50	<0.50
Carbon dioxide (CO_2) [mg.l^{-1}]	530	487	710	1630

*Temperature of sample P reservoir according to reference laboratories PLZ (http://www.rlplz.cz/index.htm).

Table S2: Mock community composition.

Microorganism	Strain
Rhodococcus jostii	RHA1
Pseudomonas alcaliphila	JAB1
Pseudomonas putida	JB
Rhizobacterium tumefaciens	C58
Pseudarthrobacter chlorophenolicus	A6
Achromobacter xylosoxidans	A8
Pandoraea pnomenusa	B356
Paraburkholderia xenovorans	LB400
Micrococcus luteus	NCTC2665
Pseudomonas stutzeri	JM300
Bacillus pumilus	SAFR032
Methylobacterium radiotolerans	JCM2831
Cupriavidus necator	H850
Escherichia coli	DH5α
Deinococcus radiodurans	R1

Table S3: List and composition of the used media.

Medium	Composition
10R2A	1,8% Noble agar, 1/10 Reasoner's 2A medium , thermal water filtrate of corresponding water spring
AF	1,8% Noble agar, thermal water filtrate of corresponding water spring
AFA	1,8% Noble agar, 0,05% acetate, thermal water filtrate of corresponding water spring
AFL	1,8% Noble agar, 0,05% lactate, thermal water filtrate of corresponding water spring
AFS	1,8% Noble agar, 0,05% succinate, thermal water filtrate of corresponding water spring
Inorg	Adapted from Soriano and Walker, 1968 (https://doi.org/10.1111/j.1365-2672.1968.tb00397.x)
TA	DSMZ catalogue number 878

Table S4: Percentage of unclassified ASVs at different taxonomic levels.

	V	M	S	P
Phylum	0.1002	0.0873	0.0673	0.0048
Class	0.1944	0.1703	0.1742	0.0192
Order	0.3759	0.3805	0.3485	0.0631
Family	0.6539	0.6109	0.5205	0.0981
Genus	0.9789	0.9567	0.8868	0.1533

Table S5: Sequencing coverage computed using package iNEXT.

iNEXT: Compare 8 assemblages with Hill number order q = 0		
**Sample Name**	**Observed Species Richness**	**Sample Coverage**
V_autumn	542	0.9989
V_spring	532	0.9990
M_autumn	923	0.9969
M_spring	875	0.9993
S_autumn	1357	0.9988
S_spring	1475	0.9989
P_autumn	623	0.9990
P_spring	520	0.9993

Table S6: Genome statistics of the phylogenetically novel bacterial species and results from the annotation server PATRIC.

	***Thermomonas* sp. S9**	***Paenibacillus* sp. P25**	***Paenibacillus* sp. P26**	***Cellulomonas* sp. P24**
Number of reads (bp)	4222	164073	60396	213334
Longest read length (bp)	116973	63695	69254	73783
Average read length (bp)	13677	5119	5983	5896
Total number of bases	57746839	840029150	361352134	1257844743
Contigs	2	1	1	2
Genome Length	2846392	8483018	8450263	4424939
GC Content	69.72	54.00	54.02	71.26
Completeness (%)	92.4	91.42	90.42	98.94
Contamination (%)	1.06	1.22	1.22	0.19
PATRIC CDS	2983	11369	11634	4277
Proteins with functional assignments	1999	6343	6512	2561
CRISPR array	0	0	0	1
CRISPR repeat	0	0	0	63
CRISPR spacer	0	0	0	62
Antibiotic Resistance (PATRIC)	33	76	77	31

Table S7: Functional categories distribution in the genomes of the phylogenetically novel bacterial species.

**Functional categories**	***Thermomonas* sp. S9**	***Paenibacillus* sp. P25**	***Paenibacillus* sp. P26**	***Cellulomonas* sp. P24**
Metabolism	403	1198	1286	569
Energy	237	286	294	256
Protein Processing	226	255	264	210
Cellular Processes	97	261	282	73
Stress Response, Defense, Virulence	88	169	172	95
DNA Processing	85	101	120	84
RNA Processing	76	71	77	43
Membrane Transport	56	69	64	71
Cell Envelope	33	15	16	20
Miscellaneous	23	15	16	4
Regulation and Cell Signaling	6	15	12	16

Figure S8: UBCG-based core-genome phylogenetic analysis depicting the distinct positioning of strain S9 with members of the genus *Thermomonas*. Bootstrap values (expressed as percentages of 1000 replications) of above 70% are shown at the branch points.

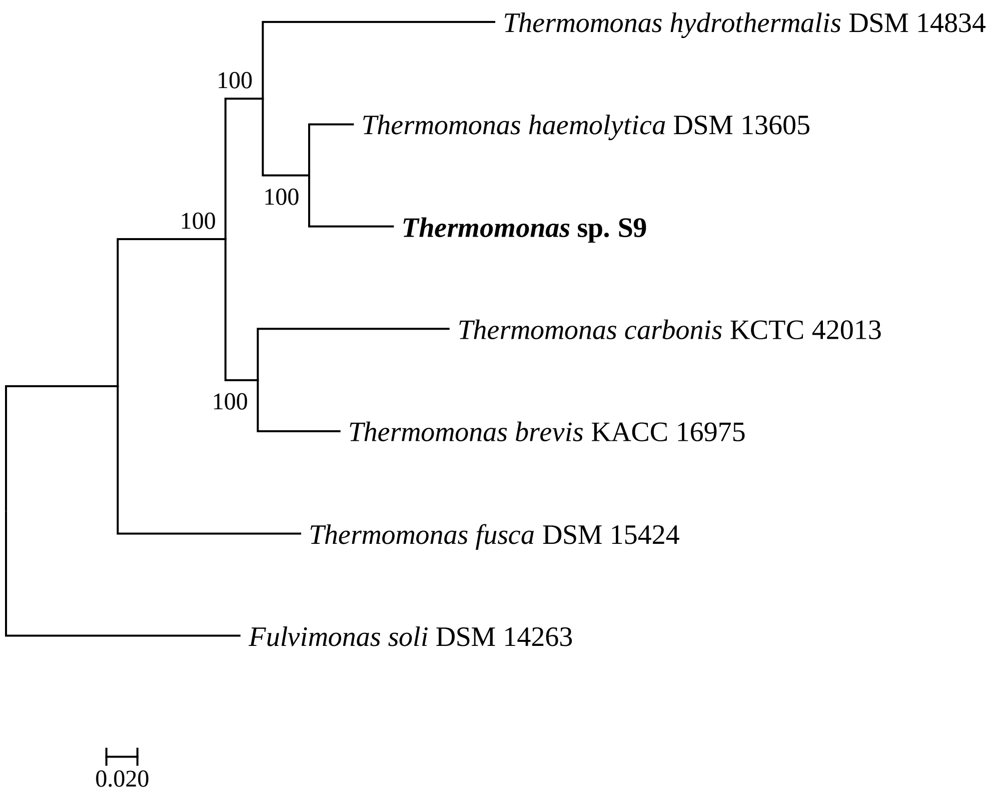

Figure S9: UBCG-based core-genome phylogenetic analysis depicting the distinct positioning of strains P25 and P26 with members of the genus *Paenibacillus*. Bootstrap values (expressed as percentages of 1000 replications) of above 70% are shown at the branch points.

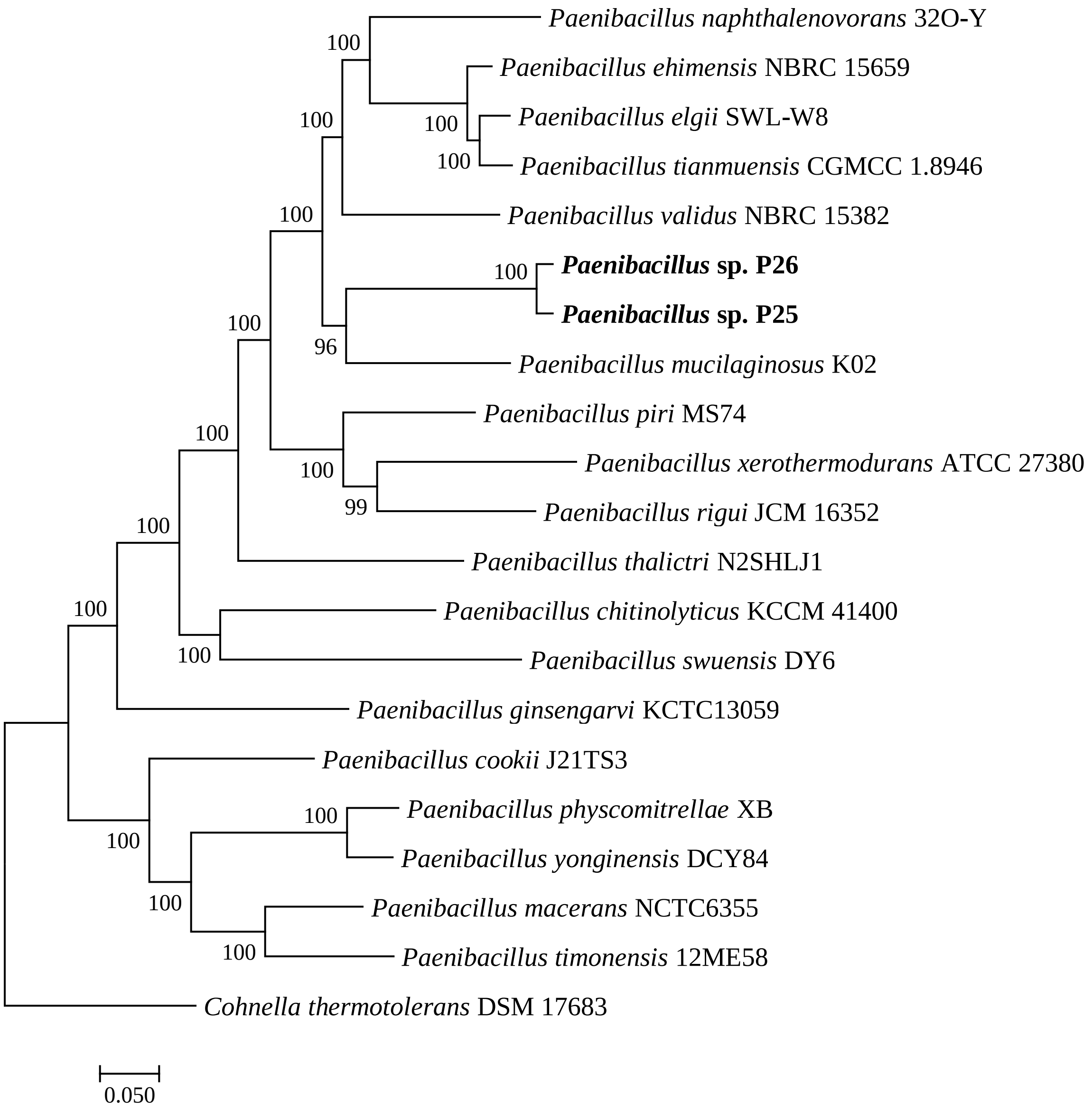

Figure S10: UBCG-based core-genome phylogenetic analysis depicting the distinct positioning of strain P24 with members of the genus *Cellulomonas*. Bootstrap values (expressed as percentages of 1000 replications) of above 70% are shown at the branch points.

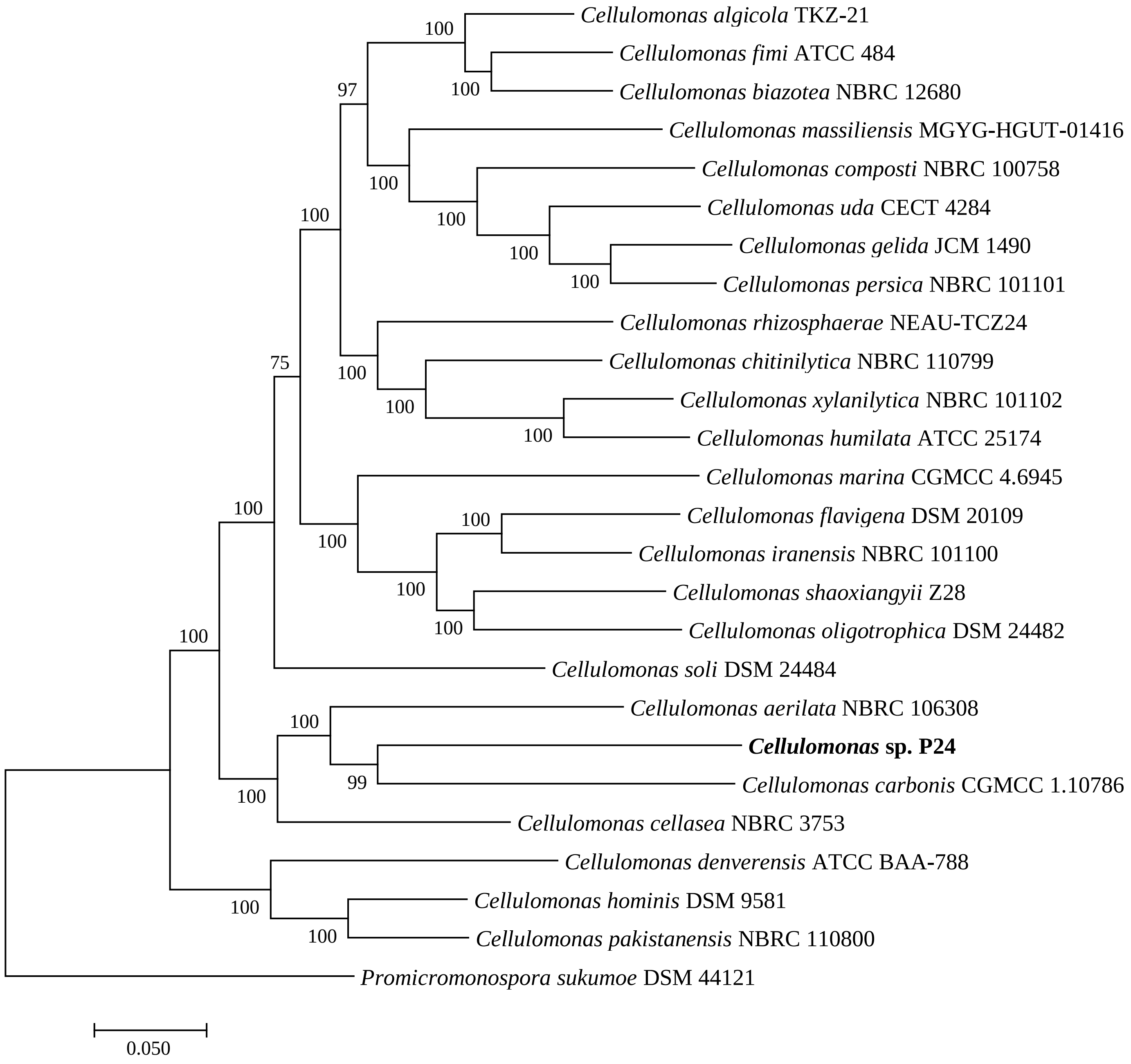

Figure S11: Differences in KEGG categories abundances in the genomes of strains P25 and P26.

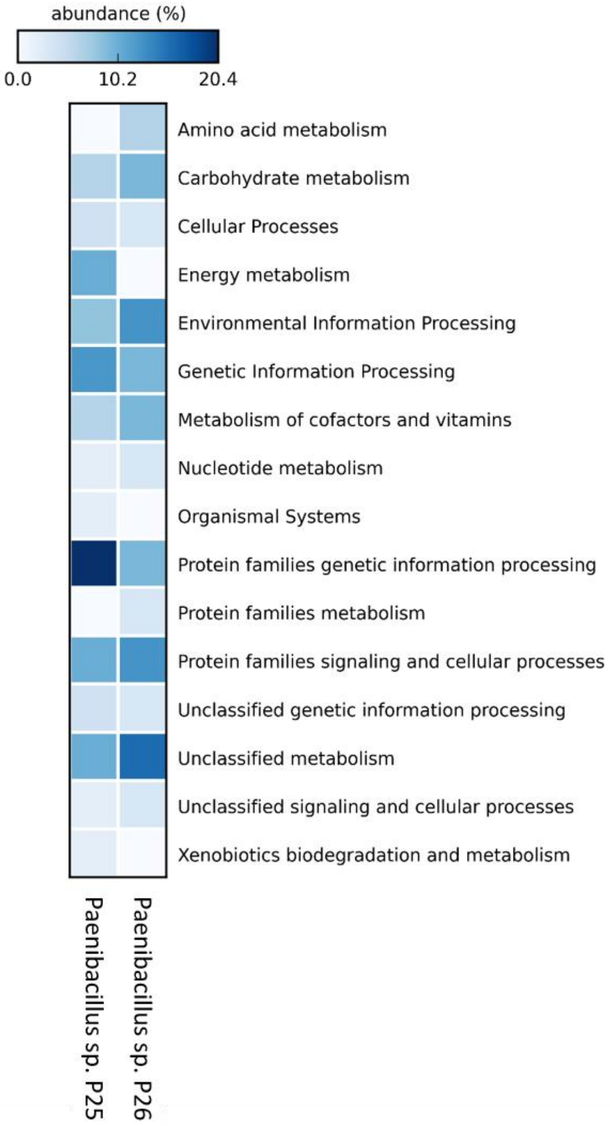

Supplement: Supplementary file 1 — Additional file 1. Table S1: Chemical composition, temperature, and flow rates of the examined thermal springs. Table S2: Mock community composition. Table S3: List and composition of the used media. Table S4: Percentage of unclassified ASVs at different taxonomic levels. Table S5: Sequencing coverage computed using package iNEXT. Table S6: Genome statistics of the phylogenetically novel bacterial species and results from the annotation server PATRIC. Table S7: Functional categories distribution in the genomes of the phylogenetically novel bacterial species. Figure S8: UBCG-based core-genome phylogenetic analysis depicting the distinct positioning of strain S9 with members of the genus Thermomonas. Bootstrap values (expressed as percentages of 1000 replications) of above70% are shown at the branch points. Figure S9: UBCG-based core-genome phylogenetic analysis depicting the distinct positioning of strains P25 and P26 with members of the genus Paenibacillus. Bootstrap values (expressed as percentages of 1000 replications) of above 70% are shown at the branch points. Figure S10: UBCG based core-genome phylogenetic analysis depicting the distinct positioning of strain P24 with members of the genus Cellulomonas. Bootstrap values (expressed as percentages of 1000 replications) of above 70% are shown at the branch points. Figure S11: Differences in KEGG categories abundances in the genomes of strains P25 and P26. [file 40793_2022_440_MOESM1_ESM.pdf]
